# Detection of Circulating Tumor Cells Using Negative Enrichment Immunofluorescence and an In Situ Hybridization System in Pancreatic Cancer

**DOI:** 10.3390/ijms18040622

**Published:** 2017-03-23

**Authors:** Yu Xu, Tai Qin, Jing Li, Xiuchao Wang, Chuntao Gao, Chao Xu, Jihui Hao, Jingcheng Liu, Song Gao, He Ren

**Affiliations:** Department of Pancreatic Cancer, Key Laboratory of Cancer Prevention and Therapy, National Clinical Research Center for Cancer, Tianjin Medical University Cancer Institute and Hospital, Tianjin 300060, China; happyxuyu@gmail.com (Y.X.); qintai.doctor@gmail.com (T.Q.); lijing20171607@gmail.com (J.L.); wangxiuchao@tjmuch.com (X.W.); gaochuntao@tjmuch.com (C.G.); XC2008092115@gmail.com (C.X.); haojihui@tjmuch.com (J.H.); liujingcheng@tjmuch.com (J.L.); foxgao2017@gmail.com (S.G.)

**Keywords:** triploid, circulating tumor microembolus, NE-iFISH, pancreatic cancer

## Abstract

Pancreatic cancer (PC) is the most lethal type of gastrointestinal cancer, and early detection and monitoring is an urgent problem. Circulating tumor cells (CTCs) are emerging as a non-invasive biomarker for tumor detection. However, the low sensitivity is a main problem in the traditional CellSearch System for detecting CTCs, especially in patients with PC. In this study, we used negative enrichment (NE), immunofluorescence and in situ hybridization (FISH) of chromosome 8 (NE-iFISH) to capture and identify CTCs in PC patients. We showed that the NE-iFISH system exhibited a dramatically high detection rate of CTCs in PC patients (90%). The diagnostic rate of PC reached 97.5% when combining CTCs ≥ 2 and carbohydrate antigen 19-9 (CA19-9) > 37 µmol/L. The 1-year survival in the group of CTCs < 3 was significantly higher than that of CTCs ≥ 3 (*p* = 0.043). In addition, we analyzed the role of chromosomal instability in CTCs detection. The group of triploid (three hybridization signals of chromosome 8) CTCs ≥ 3 showed a shorter 1-year survival (*p* = 0.0279) and overall survival (*p* = 0.0188) than the group with triploid CTCs < 3. Importantly, the triploid CTC number but not the overall CTC counts could be a predictor of chemo-sensitivity. Moreover, circulating tumor microembolus (CTMs) were found in stage IV patients, and were positively related to the poor response to chemotherapy. In conclusion, the NE-iFISH system significantly improved the positive detection rate of CTCs and triploid CTC could be used to predict prognosis or the response to the chemotherapy of PC patients. CTM is a potential indicator of the chemotherapeutic effect in advanced PC patients.

## 1. Introduction

Pancreatic cancer (PC) is regarded as a lethal malignancy with an extremely low 5-year survival rate (<5%) [1,2]. Due to the hidden nature of the symptoms, early diagnosis is still a challenge in the treatment of pancreatic cancer, and only 15%–20% patients with pancreatic cancer are considered to have resectable disease when first diagnosed [3]. At present, the gold standard of diagnosis is still the pathological test in which ultrasound-guided fine-needle biopsy is commonly used to obtain tissue specimens. However, the invasive procedure may result in unexpected complications, including infection, bleeding, pancreatic fistula, bile leakage, etc. Besides, the sensitivity of imaging-guided biopsy tests for pancreatic cancer is only 80% [2] due to epithelial contamination or tumor dislocation. Currently, carbohydrate antigen 19-9 (CA19-9) is the most popular biomarker and has been commonly used as a prognostic indicator in pancreatic cancer patients [4,5]. However, the sensitivity and specificity of CA19-9 for diagnosing pancreatic cancer are still in doubt. It was reported that pancreatic benign diseases, chronic pancreatitis and cholangitis can also lead to the increase in CA19-9 levels [6]. In contrast, PC patients with a negative Lewis antigen might possess a normal CA19-9 level. Therefore, there is an urgent need to find a sensitive and specific non-invasive diagnostic approach for PC.

Circulating tumor cells (CTCs) are the tumor cells that fall off from solid tumor masses and travel into the peripheral blood circulation, and they have been popularly detected by the CellSearch system and used as promising biomarkers to monitor chemotherapeutic efficacy in prostate cancer, breast cancer and colorectal cancer [7,8,9]. The detection of CTCs by the CellSearch system is dependent on the expression of epithelial cell adhesion molecule (EpCAM) and cytokeratins (CK) on the surface of CTCs. However, the expression of EpCAM and some CKs in CTCs is significantly weakened during the process of epithelial to mesenchymal transition (EMT) in PC [10,11]. Thus, CTCs could be missed by the CellSearch system, in which the detection rate of CTCs is low [12,13]. Currently, it is reported that negative enrichment combined with immunofluorescence and in situ chromosomal hybridization (NE-iFISH) to detect CTCs is effective in advanced gastric cancer [14]. Since this system is independent of the expression of epithelial marker of CTCs, it would be reasonable to introduce it into the detection of CTCs in PC patients. Aneuploidy is the most common hallmark of human solid tumors and is mostly caused by chromosomal instability (CIN). The replication stress induced by the gain of chromosome in the nucleus may lead to genomic instability (GIN) which contributes to tumorigenesis [15,16,17,18]. Aneuploidy is positively associated with inherent or acquired chemotherapeutic drug resistance [15,19,20,21]. The aneuploidy was reported as an independent marker for the recurrence of colon cancer [22]. More importantly, it was also reported that aneuploidy in CTCs was associated with poor overall survival and progression-free survival in lung cancer, breast cancer, ovarian cancer and oral cancer [22,23,24].

In our study, we used the NE-iFISH system to measure aneuploidy in CTCs from PC patients and dynamically monitored CTCs during the process of chemotherapy in PC patients. We also explored the sensitivity and the specificity of the combination of CA19-9 and CTCs determined by the NE-iFISH system in the diagnosis of pancreatic cancer.

## 2. Results

### 2.1. Patient Characteristics

The characteristics of the 40 PC patients and 43 control cohort included in this study are shown in Table 1. In the PC patient cohort, there were 25 males and 15 females; the median age at diagnosis was 59 years of age (62 years of age for males and 58 years of age for females). Twenty-four tumor masses were located at the head of pancreas and 16 samples located in the body and tail of pancreas. Patients were divided into two clinical groups: (1) Eleven patients with resectable cases (4 patients as Stage І and 7 patients as stage II); (2) Twenty-nine patients with a locally advanced disease (5 patients as Stage III) or metastatic lesions (24 patients as Stage IV). The control group consisted of 43 individuals, including 35 healthy donors and 8 patients with pancreatic benign diseases (3 as cystadenocarcinoma, 1 as autoimmune pancreatitis, 3 as pancreatic intraductal papillary mucinous neoplasms and 1 as acute pancreatitis). Due to the benign nature of the pancreatic lesion, 8 patients were included into control cohort.

### 2.2. Stability Test of Electronic Microscope

**Figure 1:**
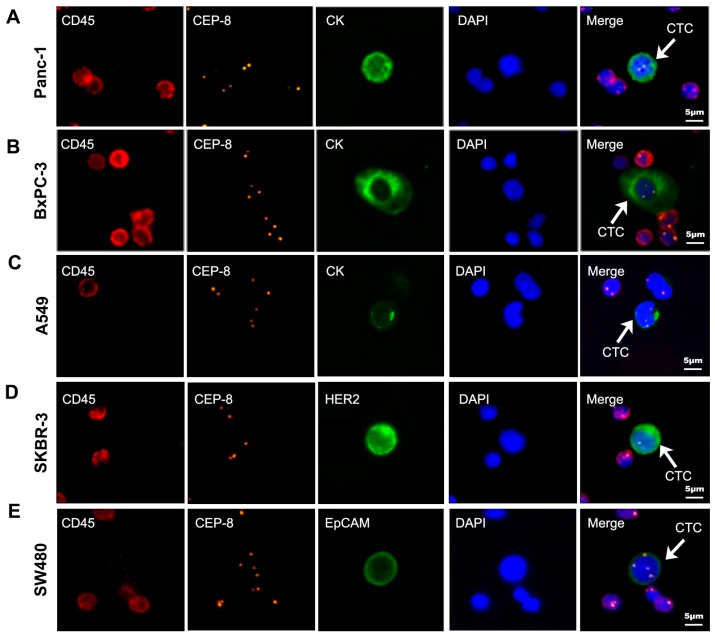
Five cell lines identified by negative enrichment combined with immunofluorescence and in situ chromosomal hybridization (iFISH; white arrow) DAPI: blue; CD45: red; CEP8: orange; CK18: green; HER2: green; EpCAM: green. (**A**) CK18 + Panc-1 cell; (**B**) CK18 + BxPC-3 cell; (**C**) CK18 + A549 cell; (**D**) HER2 + SKBR-3 cell; (**E**) EpCAM + SW480 cell. Scale bar = 5 µm. CEP-8: chromosome enumeration probes 8; DAPI: 4′,6-diamidino-2-phenylindole; CK18: cytokeratins 18; CD45: cluster of differentiation 45; HER2: human epidermal growth factor receptor-2; EpCAM: epithelial cell adhesion molecule; CTC: circulation tumor cell.

Five cell lines (Bxpc-3, PANC-1, A549, SW480, SK-BR3) were used to evaluate the stability of this electronic microscope. Evaluation of the result was performed by experienced technicians; three times consecutive parallel judgement of the result was also defined using the microscope (Table 2). Cells were detected by the NE-iFISH system and identified by the automatic microscope (Figure 1). The result showed that CTC counting by using the electronic microscope was more stable, objective and repeatable than counting artificially.

### 2.3. Definition of Circulating Tumor Cells *(CTC)* by Negative Enrichment Immunofluorescence and an In Situ Hybridization (NE-iFISH) System

**Figure 2:**
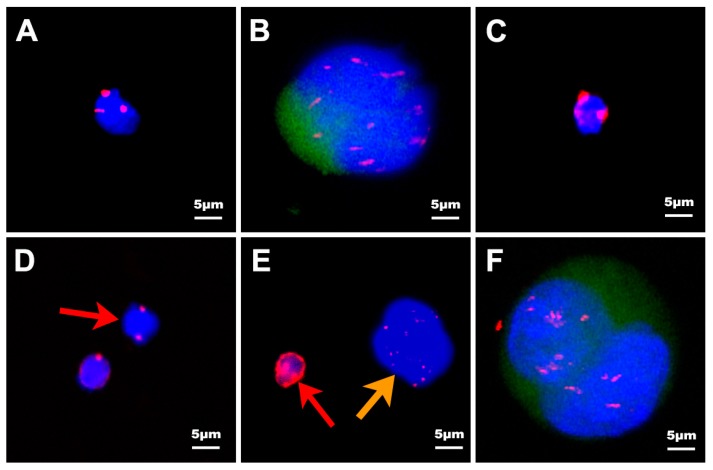
In situ hybridization with chromosome 8 and CK18, CD45, DAPI immunofluorescence of CTCs, CTMs and white blood cells (WBCs) enriched from pancreatic cancer patients. DAPI: blue; CD45: red; CEP8: red; CK18: green. (**A**) CK18−, CD45−, DAPI+, CEP-8 = 3; (**B**) CK18+, CD45−, DAPI+; (**C**) CK18−, CD45+, DAPI+; (**D**) CK18−, CD45−, DAPI+, CEP-8 = 2, (red arrow); (**E**) CK18− CTMs (orange arrow). WBCs stained with weak CD45 are indicated by the red arrow; (**F**) CK18+ CTMs. Scale bar = 5 µm.

**Figure 3:**
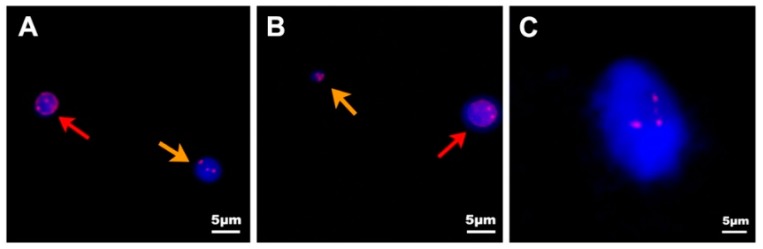
Different size of CTCs isolated by the NE-iFISH system. DAPI: blue; CD45: red; CEP8: red. (**A**) The size of WBC-like CTCs (orange arrow) is similar to that of the WBCs (red arrow) stained with CD45; (**B**) The size of tiny CTCs (orange arrow) is much smaller than that of WBCs (red arrow) stained with weak CD45; (**C**) The size of large CTCs is greater than 15 µm. Scale bar = 5 µm.

**Figure 4:**
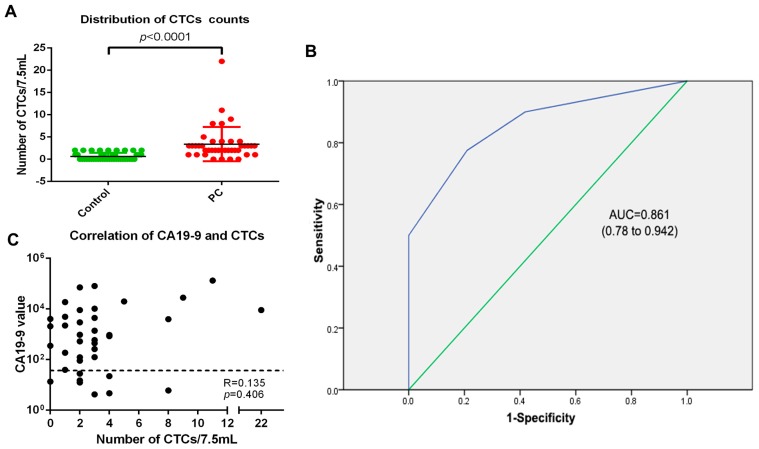
The application of CTCs in the diagnosis of PC. (**A**) The distribution of CTC numbers in the PC patients and control cohort was statistically significant (*p* < 0.001). Data was plotted with mean ± S.D; (**B**) The receiver operator characteristic (ROC) curve was used to determine the cut-off value of CTCs between pancreatic cancers and pancreatic benign disease as well as healthy donors. When the cut-off value was 1.5 cells/7.5 mL, the sensitivity of CTCs for diagnosis of pancreatic cancer was 77.5%, while the specificity was 79.1%. (AUC = 0.861, 95% CI 0.78–0.942, *p* < 0.0001); (**C**) Relevance between the number of CTCs and CA19-9 value (R = 0.135, *p* = 0.406). The black dashed line represented the CA19-9 threshold of 37 U/mL. S.D: standard deviation; AUC: area under roc curve; CI: confidence interval.

The immunostaining markers used for distinguishing different circulating tumor cells were cytokeratins 18 (CK18), cluster of differentiation 45 (CD45), 4′,6-diamidino-2-phenylindole (DAPI) and chromosome enumeration probes 8 (CEP-8) as described in Material and Methods. White blood cells were stained with CD45+, DAPI+ but CTCs were stained with CD45− DAPI+ due to the specific marker on the surface of white blood cells. Cells detected in our study in PC patients can be divided into five categories in terms of the iFISH results: (1) type-A phenotype: CK18−, CD45−, DAPI+, CEP-8≠2 (Figure 2A); (2) type-B phenotype: CK18+, CD45−, DAPI+ (Figure 2B); (3) type-C phenotype: CK18−, CD45+, DAPI+ (Figure 2C); (4) type-D phenotype: CK18−, CD45−, DAPI+, CEP-8 = 2 (Figure 2D, red arrows); (5) type-E phenotype: circulating tumor microemboli (CTMs) (Figure 2E, orange arrows, 2F). Because of the lack of the expression of CK and the heteroploidy of the cells, type-A cells were confirmed as CTCs. Type-B cells were exhibited as CK positive, so they were also confirmed as CTCs. Only two pancreatic benign cases (1 pancreatic cystadenoma patient and 1 pancreatic intraductal papillary mucinous neoplasm (IPMN) patient) in 83 donators were detected as type-B cells. Type-C cells were CK18−, CD45+, DAPI+, no matter how many probe signals of CEP-8, and these cells were confirmed as white blood cells (WBCs). About 10^4^–10^5^ type-C cells were detected in every enriched slide. For type-D cells it was uncertain whether they belonged to CTCs or hematopoietic cells, since they were human nature diploids but without CK18 staining. Therefore, these cells were considered as indeterminate cells, and we did not add these cells into the classes of CTCs in our study. Only 0–5 type-D cells of each patient were detected in our study. Type-E cells were circulating tumor microemboli (CTMs) gathered by more than one CTC. CTM were classified into two classes, CK18− (Figure 2E, orange arrows) and CK18+ (Figure 2F), respectively. In conclusion, type-A, type-B and type-E were classified as CTCs. Type-C was classified as hematopoietic cells and type-D as indeterminate cells.

The size of CTCs was generally 15–30 µm larger than hematopoietic cells [25]. In our study, the size of CTCs range from 2–4 to 70–80 µm. Based on the size, CTCs can be divided into three categories: (1) tiny CTCs: the maximum diameter of CTCs less than 5 µm (Figure 3B, orange arrows); (2) WBC-like CTCs: the maximum diameter of CTCs range from 5 to 15 µm (Figure 3A, orange arrows); and (3) large CTCs: the maximum diameter of CTCs more than 15 µm (Figure 3C). The total number of CTCs detected by NE-iFISH system in our research was 416, including 8 (1.92%) tiny cells, 99 (23.8%) WBC-like cells and 297 (74.28%) large CTCs.

CTCs were detected in 12/35 healthy donators (34.28%, with the median of 0, range from 0 to 2), 6/8 pancreatic benign disease donators (75%, with the median of 2, range from 0 to 2) and 36/40 PC patients (90%, with the median of 2, range from 0 to 22). The difference between PC patients and the control cohort was statistically significant (*p* < 0.0001) (Figure 4A). PC patients exhibited more CTCs than pancreatic benign disease patients and healthy people. The receiver operator characteristic (ROC) curve was used to reveal the sensitivity and specificity, which was 77.5% and 79.1%, respectively. Therefore, CTCs could be a biomarker to help diagnosing pancreatic cancer when the cut-off value was decided as 1.5 CTCs/7.5 mL (AUC = 0.861, 95% CI 0.78–0.942, *p* < 0.0001) (AUC: Area Under roc Curve; CI: Confidence Interval) (Figure 4B). The cut-off value defined by the ROC curve was 1.5 CTCs/7.5 mL, but the counts of CTCs are integral, so the nearby one or two CTCs/7.5 mL were considered as the cut-off value. However, when the cut-off value was defined as one CTC/7.5 mL, the specificity of CTCs was only 58.1%. This was too low for clinical examination, so we defined two CTCs/7.5 mL as the cut-off value. Thirty-one of the 40 PC patients (77.5%) were judged as CTC-positive and 31/40 (77.5%) patients were judged as being CA19-9-positive. Since 39 cases (97.5%) presented either being CA19-9- or CTC-positive, the combination of CA19-9 and CTCs would vastly improve the detection rate of pancreatic cancer patients. In addition, CTC counts and CA19-9 values were relatively independent parameters, as the Spearman correlation (R) was 0.135 (*p* = 0.406) (Figure 4C).

### 2.4. Clinical Significance of CTCs in PC

**Figure 5:**
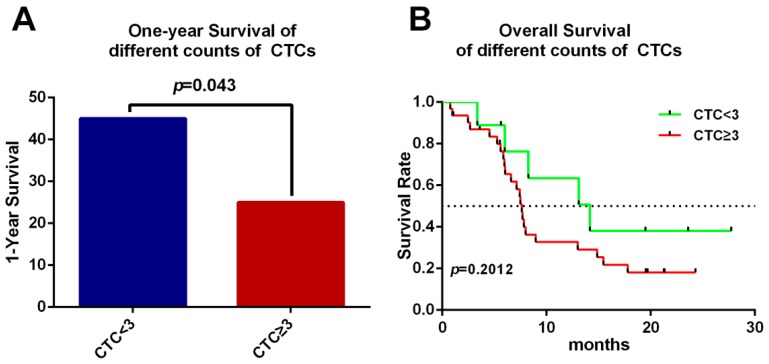
Correlation between CTCs and prognosis. (**A**) There was a significant decrease of 1-year survival in the CTC ≥ 3 group compared with the CTC < 3 group (hazard ratio for death, 0.405; 95% CI 0.1738 to 0.9709 *p* = 0.043); (**B**) Overall survival is irrelevant to the number of CTCs; the CTC < 3 group showed a median of 14.17 months versus the 7.63 months of CTC ≥ 3 group. (Hazard ratio for death, 0.5393; 95% CI 0.2579 to 1.326 *p* = 0.2012). The black dashed line indicates the median at which 50% of patients were alive.

There was no significant difference in age, gender, TNM (Tumor, Node, Metastasis) stages, location of the tumor, nor CA19-9 between the two groups (Supplementary Table S1). To evaluate the impact of the cut-off value of CTC count on the hazard ratio of survival, CTC numbers of 2, 3 and 4 per 7.5 mL blood were tested for differentiation with 1-year survival and overall survival by Kaplan–Meier method. We have found that when the cut-off value of CTCs was set at 3, the 1-year survival was discrepant between these two groups. The CTC < 3 group displayed significantly increased 1-year survival than CTC ≥ 3 group analyzed by log-rank test (hazard ratio for death, 0.405; 95% CI 0.1738 to 0.9709 *p* = 0.043) (Figure 5A). However, there was no relationship between overall survival (OS) and the number of CTCs (hazard ratio for death, 0.5393; 95% CI 0.2579 to 1.326 *p* = 0.2012) (Figure 5B).

**Figure 6:**
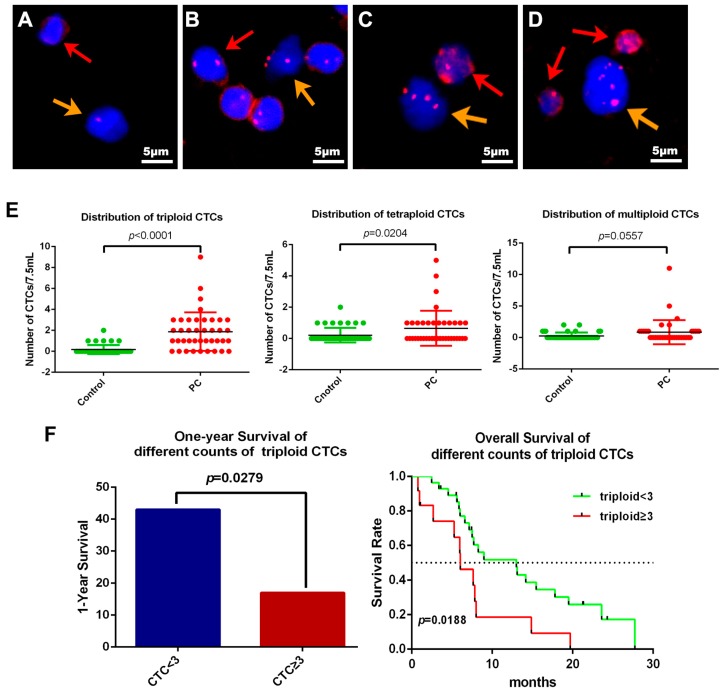
Correlation between the aneuploidy of CTCs and the prognosis. DAPI: blue; CD45: red; CEP8: red; CK18: green. (**A**) A monoploid CTC (orange arrow) and WBC (red arrow) isolated from one PC patient; (**B**) A non-hematopoietic triploid CTC is indicated by the orange arrow. A diploid WBC stained with weak CD45 is marked by the red arrow; (**C**) A tetraploid CTC is indicated by the orange arrow and the other one stained by CD45 is a WBC; (**D**) A multiploid CTC is indicated by the orange arrow. The other two cells stained by CD45 were WBCs; (**E**) The distribution of triploid and tetraploid numbers in the control group and PC group were statistically significant (*p* < 0.001, *p* = 0.0204), but not in the multiploid group (*p* = 0.0557); (**F**) One-year survival was significantly relevant to the counts in the triploid group (hazard ratio for death, 0.3941; 95% CI 0.1144 to 0.8783 *p* = 0.0279). The triploid CTC < 3 group had a median overall survival of 13 months longer than the triploid CTC ≥3 group (6.03 months; hazard ratio for death, 0.4309; 95% CI 0.1374 to 0.8219 *p* = 0.0188). The black dashed line indicates the median. Scale bar = 5 µm.

Based on the CEP-8, CTCs can be classified as monoploidy (3.8%, 16 of 416 CTCs) (Figure 6A), triploidy (28.85%, 120 of 416 CTCs) (Figure 6B), tetraploidy (10.58%, 44 of 416 CTCs) (Figure 6C), multiploidy (53.37%, 222 of 416 CTCs) (Figure 6D) and as CTMs (2.88%, 12 of 416 CTCs) (Figure 2E, orange arrows, 2F). In order to explore the differences in aneuploidy between the control cohort and PC patients, chromosomal instability of eight was analyzed in our study. The difference in triploid and tetraploid CTCs was statistically significant between the two groups (*p* < 0.0001, *p* = 0.0204), but there was no significant difference in multiploid CTC between these two groups (*p* = 0.0557) (Figure 6E). Further investigation to verify the correlation between aneuploid CTC and prognosis was also illustrated. Patients with triploid CTCs < 3 had an increased one-year survival compared with patients with triploid CTCs ≥ 3 (hazard ratio for death, 0.3941; 95% CI 0.1144 to 0.8783 *p* = 0.0279). Furthermore, the triploid CTCs < 3 group also had higher overall survival than the triploid CTC ≥ 3 group, with a median of 13 months versus 6.03 months (hazard ratio for death, 0.4309; 95% CI 0.1374 to 0.8219 *p* = 0.0188) which suggests that triploid CTCs may predict the prognosis of pancreatic cancer patients (Figure 6F). However, the differences in tetraploid or multiploid CTCs were not related to patients’ overall survival (Supplementary Figure S1).

### 2.5. Dynamically Monitored CTC Counts and Aneuploidy in PC Patients

**Figure 7:**
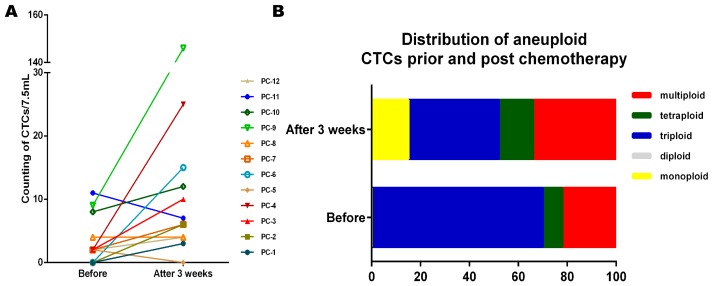
Variation of CTC count and aneuploidy prior and post chemotherapy. (**A**) CTCs of 12 metastatic patients were dynamically monitored prior and after chemotherapy. PC-1~PC-12 are the codes of 12 metastatic pancreatic ductal adenocarcinoma patients, respectively. CTC numbers for PC-1, PC-2, PC-3, PC-4, PC-6, PC-7, PC-9, PC-10, PC-12 were obviously increased after chemotherapy, especially for PC-4, and PC-9. CTC numbers for PC-5, PC-11 were slightly decreased after chemotherapy. CTC numbers for PC-8 exhibited a stable tendency prior to and after chemotherapy; (**B**) The proportion of triploid CTC prior- and post-chemotherapy was statistically significant (χ^2^ = 30.381, *p* < 0.001). PC-9 presented numerous multiploid CTCs (146 CTCs/7.5 mL) after 3 weeks of chemotherapy, which spread the distribution of the CTCs, therefore we rejected PC-9 in this study. CTC: circulating tumor cells.

Among the 40 PC patients, we dynamically monitored 12 metastatic pancreatic ductal adenocarcinoma patients (PC-1~PC-12) (recruited by ABI-007-PANC-001, Safety and Efficacy Study of Nab-paclitaxel Plus Gemcitabine in Chinese Patients with Metastatic Pancreatic Cancer, NCT02017015). A total of 7.5 mL peripheral blood was collected to detect CTCs by NE-iFISH before patients received chemotherapy, as well as 3 weeks (1 cycle) after the chemotherapy. Computed tomography (CT) evaluation was also performed 6 weeks after the chemotherapy. After evaluating the tumor stage by Response Evaluation Criteria in Solid Tumors (RECIST) 1.0 standard, four kinds of outcomes appeared in our research, including complete response (CR; 1/12), partial response (PR; 5/12), stable disease (SD; 1/12) and progressive disease (PD; 5/12). After receiving gemcitabine based chemotherapy, the CTC numbers detected in most patients were elevated, except for PC-5, PC-11 with a slight reduction and PC-8 which were without change (Figure 7A). This result indicated that the number of CTCs could not be regarded as an indicator for chemotherapy responses. However, the proportion of triploid detected by the NE-iFISH was significantly decreased after chemotherapy (χ^2^ = 30.381, *p* < 0.001) (Figure 7B).

### 2.6. CTM Indicates Chemo-Resistance and a Poor Prognosis

**Figure 8:**
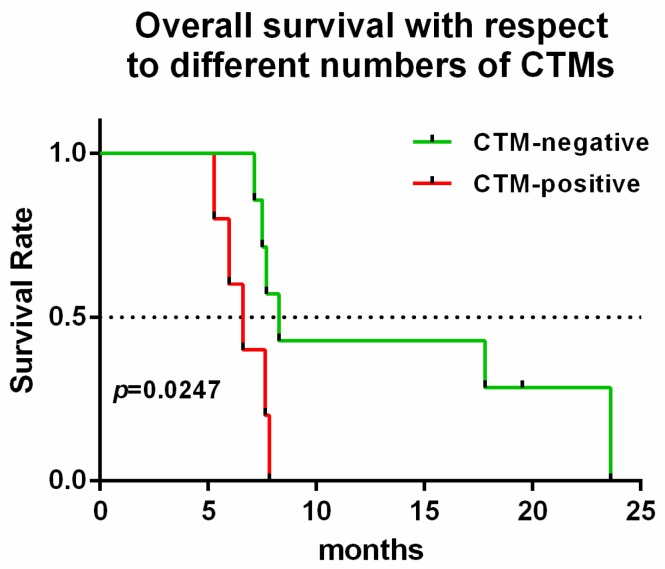
Correlation between CTM and prognosis. There was significantly longer overall survival in the CTM-negative group compared to the CTM-positive group, with the median of 8.27 months versus 6.6 months. (hazard ratio for death, 0.3008; 95% CI 0.03755 to 0.7191 *p* = 0.0247). The black dashed line indicates the median.

CTM was detected in 6 out of 40 patients and they were all diagnosed as metastatic PC (stage IV). Among them, PC-24 rejected the follow-up examination, so we did not acquire the CTC and CTM information after chemotherapy. The information of those five patients is shown in Table 3. Interestingly, those five patients detected with CTM were all evaluated as PD by regular CT evaluation after chemotherapy. In addition, their CA19-9 level was not all parallel to the CT evaluation. Among the five patients, PC-8 exhibited negative CA19-9 values prior and after chemotherapy, so the CA19-9 value cannot be used to evaluate this disease in PC-8. Twelve PC patients (recruited by ABI-007-PANC-001 phase 2 multi-center study) were divided into two groups of CTM, CTM negative and CTM positive. Survival analysis showed that the CTM-negative group exhibited significantly longer survival than the CTM-positive group, with the median of 8.27 months versus 6.6 months (*p* = 0.0277) (Figure 8), suggesting CTM could be a prognostic marker.

## 3. Discussion

Due to the risk of fine needle biopsy, there was an urgent need for a new type biomarker for tumor screening and diagnosis. CTCs, as a non-invasive, real-time blood biomarker, have received attention in cancer research. The clinical significance of CTCs has been proven in various solid tumors, such as prostate cancer, breast cancer, gastric cancer, small-cell lung cancer [26,27,28,29,30]. The American Society of Clinical Oncology (ASCO) had approved CTCs as a biomarker in metastatic breast cancer, prostate cancer and colorectal cancer [31]. However, because of the limited quantity of CTCs in blood circulation (1 out of a billion blood cells) [32], an efficient method to isolate and identify CTCs became necessary.

The CellSearch system, as an approved method to test CTCs, depends on the expression of EpCAM and greatly limited the positive rate of CTCs in some epithelium-originated solid tumors like breast cancer and pancreatic cancer [33,34] for the reason that the expression of EpCAM was reduced during EMT. It has already been shown that a very low positivity (11%) was reported by CellSearch system in patients with locally advanced pancreatic cancer [35]. We also tested the positive rate of CellSearch system and NE-iFISH system in one PC patient (PC-10) in our study. With a total volume of 7.5 mL of peripheral blood, CTCs were enriched and identified using CellSearch system and NE-iFISH system at the same time, respectively. Five triploid, 7 multiploid and 1 CTM were found by NE-iFISH system, while none were found by CellSearch system. CTCs detected by NE-iFISH system also exhibited diversity in cellular size, especially for the tiny and WBC-like CTCs compared to the traditional membrane filtration system. It has been reported that tumors cells going through EMT tend to be smaller than cells without EMT, and some CTCs predict a significantly poor prognosis in gastric cancer [36]. However, there is no relationship between overall survival (OS) and small CTCs (tiny CTCs plus WBCs-like CTCs) in our study (hazard ratio for death, 0.9713; 95% CI 0.4757 to 1.975 *p* = 0.934) (Supplementary Figure S2). Therefore, NE-iFISH has a higher positive rate and broader detectable species than the CellSearch system and the traditional membrane filtration system in PC.

In most studies, CTC readings were performed by pathologists or technicians and smaller technical errors were unavoidable. The stability test of NE-iFISH was carried out by the automatic electronic microscope, which could largely eliminate the systemic error of quantification of CTCs. The stability test of the microscope was evaluated via five cell lines. A total of 7.5 mL peripheral blood was spiked with different numbers of human pancreatic cancer cells including Bxpc-3, PANC-1 and three other cell lines, A549, SW480, SK-BR3. These acted as positive controls and were processed as described in Materials and Methods. The result shows that the definition judged by the electronic microscope was greatly stable, objective and repeatable.

The positive rate in the recruited 40 PC patients detected by NE-iFISH system was 90% (36/40), and the ROC curve showed that when the cut-off value was 1.5 CTCs /7.5 mL, the sensitivity of detection for CTCs was 77.5% and the specificity was 79.1%. CTCs isolated and identified by the NE-iFISH system were judged using two methods, the aneuploidy of tumor cells and tumor antigens in CTCs (CK), respectively. NE-iFISH exhibited a high positive rate in PC (90%, 36 of 40 PC patients), so we have not attempted to use other tumor antigens to detect CTCs in this study. Further studies will identify other tumor antigens on CTCs from pancreatic ductal adenocarcinoma (PDAC) patients.

CA19-9 is the only biomarker acknowledged by the Food and Drug Administration (FDA) in evaluating surgical efficacy [28]. The sensitivity of CA19-9 for PC ranged from 62% to 87.5% [37,38,39,40,41], and it is the only marker recommended for clinical use by the National Comprehensive Cancer Network (NCCN) guidelines for PDAC. Carcinoembryonic antigen (CEA) is the second most common serum biomarker currently used clinically to detect PDAC. The sensitivity of CEA for PC ranged from 33.3% to 59.1% [38,42,43,44,45]. The panel of CA 19-9, CEA, and tissue inhibitor of metalloproteinase-1 (TIMP-1) demonstrated a sensitivity of 71% for PC [46]. The combination of CA19-9 and CEA did not mean a high positive rate for PC. We demonstrated that CTCs as a kind of liquid biopsy can be used as a diagnostic method as well as a prognostic predictor according to our data. CTCs could be categorized into different types due to the aneuploidy difference, which is more direct evidence of diagnosing PC. We found in our study that the sensitivity of CA19-9 for diagnosing pancreatic cancers reaches 77.5%. When combined with CTCs, the positive rate of diagnosis of PC could reach as high as 97.5%. Therefore, the combination of these two biomarkers will cover the majority of PC patients.

Furthermore, CTCs could provide real-time personal pathological and genetic mutation information as well. More importantly, the number of CTCs may be a predictor of prognosis for PC. In our study, 40 PC patients were divided into two groups, CTC < 3 group and CTC ≥ 3 group. The one-year survival but not overall survival was significantly different in these two groups. Moreover, the triploid CTCs < 3 group displayed significantly increased one-year survival and overall survival compared to the triploid CTCs ≥ 3 group. However, tetraploid and multiploid groups were not significantly different. It has been reported that triploid numbers were positively related to intrinsic drug resistance, whereas tetraploid and multiploid numbers may indicate acquired drug resistance [19,28]. In our study, the proportion of triploid was significantly decreased after one cycle chemotherapy, but the proportion of triploid CTC was not significantly decreased in the progression-free group (CR patients, PR patients and SD patients) (95% CI 0.1738 to 0.9709, *t* = 0.133, *p* = 0.898). This result was not statistically significant due to the small number of samples but showed that the elevation of the proportion of triploid CTCs tends to predict chemo-resistance. Cui et al. [47] showed that CTC-negative pancreatic cancer patients exhibited better progression-free survival and even a longer overall survival than CTC-positive patients, but this study is based on limited samples (*n* = 15). Yuan et al. [48] also revealed that CTC < 3 group correlated to longer overall survival than the CTC ≥ 3 group in PC. Their studies had applied solid dyed method for cell fixation (cells fixation before anti-CD45 and anti-CK18 specific binding), but we used liquid-dyed fixation (cells fixation after anti-CD45 and anti-CK18 specific binding) which achieved a tight binding of antibody to antigen.

Using this modified NE-iFISH method, we have detected CTMs. CTMs are multicellular aggregates or clusters of tumor cells. It has been reported that CTMs are associated with chemo-resistance and tumor metastasis in lung cancer [49,50] and breast cancer [51]. CTM has been examined in few cases in PC [52], and its clinical significance has not clearly reported yet. Twelve metastatic cases with pancreatic patients were dynamically monitored prior to and post-3-week chemotherapy. However, the chemotherapeutic response was not related to the change in CTCs. In addition, CTMs were all detected in patients with stage IV and they suggest a poor prognosis and chemo-resistance in advanced PC. But only a small amount of CTMs was detected in our study, a large sample size multi-center study is further needed.

In summary, The NE-iFISH system could clearly improve the detection rate of CTCs in patients with PC. If CTC enumeration is combined with the biomarker CA19-9, the positive rate of diagnosis of pancreatic cancer is nearly 97%. These non-invasive blood markers could be regarded as the mainstay of diagnosis of pancreatic cancer. Patients with detectable CTM may indicate chemo-resistance and poor survival compared to those without CTM. For patients without detectable CTM, CTC ≥ 3/7.5 mL or triploid CTC ≥ 3 predicts a poor prognosis, and the increase of the proportion of triploid after chemotherapy may forecast chemo-resistance.

## 4. Materials and Methods

### 4.1. Cell Culture

Human PC cell lines (Panc-1, BxPC-3), a lung cancer cell line (A549), a breast cancer cell line (SKBR-3) and a colon cancer cell line (SW480) were obtained from American Type Culture Collection. Cell lines (Rockville, MD, USA) were routinely cultured under standard conditions and tested for authentication at the beginning of the project. (January 2014). The cell line authentication was performed by GENEWIZ Inc. (Beijing, China) utilizing ten short Tandem Repeat (STR) loci compared with the database of the standard cell lines.

### 4.2. Patients and Sample Collection

Patients were recruited via the Department of Pancreatic Cancer at Tianjin Medical University Cancer Institute and Hospital, China between April 2014 and July 2016. The study was approved by the clinical investigation ethics committee of Tianjin Medical University Cancer Institute and Hospital (E2014092, 12 March 2014). All the patients signed the informed consent forms. The study included a total of 40 patients with pathologically confirmed PC diagnosed at different stages (resectable, locally advanced and metastatic disease). Besides, a total of 43 control donors with imaging confirmed benign tumor of the pancreas (BTP, 8 donors) or healthy control (HC, 35 donors), were also included in the study as a control cohort. After surgical treatment or chemotherapy, patients were performed with a computed tomography (CT) scan to evaluate the efficacy. Clinical response criteria were evaluated as complete response (CR), partial response (PR), stable disease (SD) and progressive disease (PD) by Response Evaluation Criteria in Solid Tumors (RECIST) 1.0 standard. A total of 7.5 mL venous blood sample was collected prior to and post treatment (1 cycle after chemotherapy) and performed within 8 h.

### 4.3. CTC Determination by NE-iFISH System

Briefly, a total volume of 7.5 mL blood was collected and centrifuged at 800× *g* for 7 min. The supernatant at the top layer was then removed for discarding all the plasma and platelets. The remaining sediment was then mixed with separation matrix (Cytelligen, San Diego, CA, USA) and then centrifuged at 450× *g* for 7 min. Immuno-magnetic beads (Cytelligen) were added to the supernatant and incubated at room temperature for 20 min to remove the majority of remaining blood cells using centrifuging at 450× *g* for 7 min and a special magnet (Cytelligen). The mixture was then centrifuged at 450× *g* for 7 min and supernatant was all collected for another centrifuge at 650× *g* for 4 min. The enriched pallets were labelled with CD45 and CK18 (EpCAM for SW480 cell lines, human epidermal growth factor receptor-2 (HER2) for SKBR3 cell lines) antibodies and then fixed on a slide at 31 °C for 16 h. A volume of 10 µL chromosome enumeration probes 8 reagent (CEP-8, Abbott, Downer’s Grove, IL, USA) was added to the center of the slide in order to cover all the cells. The cells were denatured at 76 °C for 5 min and hybridized at 37 °C for 90 min. Finally, cells were stained with 4′,6-diamidino-2-phenylindole (DAPI, Cytelligen) and then observed under the fluorescence microscope. To eliminate bias, blood collection, enrichment and immunofluorescence in situ hybridization were processed by trained and experienced technicians. The result was read by aAxioImager.Z2 microscope (Carl Zeiss Far East Co., Ltd., Oberkochen, Germany).

A total of 7.5 mL of peripheral blood was spiked with different numbers of human pancreatic cancer cells including Bxpc-3, PANC-1 and three other cell lines, A549, SW480, and SK-BR3 to obtain 100 cells per ml of blood. These acted as pancreatic cancer tumor cell-positive controls and were processed as described above. CTC counting was not only performed by practiced technicians and confirmed by an independent personnel but by Axio Imager.Z2 microscope as well. Triple counting by the microscope was carried out in our study to validate the stability of this method.

The CEP 8 Spectrum Orange DNA Probes used in our study bought from Abbott are stable and safe, and approved by the Food and Drug Administration (FDA, K953591) and CE (CONFORMITE EUROPEENNE).

### 4.4. Detection of Carbohydrate Antigen 19-9

A total volume of 3.5 mL peripheral blood was collected into tubes with Silica Clot Activator and Polymer Gel (Becton Dickinson, CA, USA) for the following steps. The carbohydrate antigen 19-9 (CA19-9) value was determined by automatic immunoassay analyzer (Modular E170, Roche, German) and auxiliary kit according to the manufacturer’s instruction. This detection was conducted in the clinical laboratory approved by ISO15189 and Bio-Rad External Quality Assurance.

### 4.5. Statistical Analysis

Statistical analysis was performed using SPSS version 18.0 software (Armonk, NY, USA).The Fisher exact test was applied for the categorical variables such as sex and stage. The sensitivity and specificity of CTCs in diagnosis of pancreatic cancer were analyzed by the receiver operating characteristics (ROC) curve. A univariate analysis was performed using the Kaplan–Meier estimate of survival to compare CTC-positive vs negative patients with the Mantel–Haenszel test. All the tests in the study are two-sided. *p* < 0.05 was recognized as statistically significant. Graph was mapped by the GraphPad Prism 6.

## 5. Conclusions

Circulating tumor cells (CTCs) could be a valuable, non-invasive surrogate marker for pancreatic cancer. The diagnostic rate of pancreatic cancer reached to 97.5% when combined CTCs ≥ 2 and CA19-9 > 37 µmol/L. Circulating tumor microembolus indicates chemo-resistance and a poor survival. CTC ≥ 3/7.5 mL or triploid ≥ 3 predict a poor prognosis, and the increase of the proportion of triploid after chemotherapy may forecast chemo-resistance.

## Figures and Tables

**Table 1:** Enumeration and characteristics of CTCs and circulating tumor microembolus (CTMs) identified by NE-iFISH System in PC patients and healthy cases.

Patients Code	Gender	Age	Location	Histological Type	Stage	Before Treatment														Treatment after 3 Weeks														Outcome (Follow Up for 28 Months)
						CA19-9 (U/ML)	CK+, CD45−, DAPI+						CK−, CD45−, DAPI+						CTM	CA19-9 (U/ML)	CK+, CD45−, DAPI+						CK−, CD45−, DAPI+						CTM	
							Monoploid	Diploid	Triploid	Tetraploid	Multiploid	Sum	Monoploid	Diploid	Triploid	Tetraploid	Multiploid	Sum			Monoploid	Diploid	Triploid	Tetraploid	Multiploid	Sum	Monoploid	Diploid	Triploid	Tetraploid	Multiploid	Sum		
PC-1	M	65	H	PDAC	IV	4050	0	0	0	0	0	0	0	/	0	0	0	0	0	934.8	0	0	0	0	0	0	0	/	2	0	1	3	0	Died
PC-2	M	51	BT	PDAC	IV	13.59	0	0	0	0	0	0	0	/	0	0	0	0	0	13.59	0	0	0	0	0	0	2	/	3	1	0	6	0	Died
PC-3	F	55	BT	PDAC	IV	9080	0	0	0	0	0	0	0	/	1	0	1	2	0	10361	0	0	0	0	0	0	0	/	0	5	5	10	0	Died
PC-4	M	54	BT	PDAC	IV	28.12	0	0	0	0	0	0	0	/	2	0	0	2	0	727.9	0	0	0	0	0	0	13	/	10	1	1	25	0	Died
PC-5	M	39	H	PDAC	IV	527.7	0	0	0	0	0	0	0	/	1	0	1	2	0	423.2	0	0	0	0	0	0	0	/	2	0	0	2	0	Died
PC-6	M	36	BT	PDAC	IV	591.4	0	0	0	0	0	0	0	/	2	1	0	3	0	1700	0	0	0	0	0	0	0	/	1	0	14	15	0	Died
PC-7	M	62	H	PDAC	IV	964.4	0	0	0	0	0	0	0	/	2	0	0	2	0	155	0	0	0	0	0	0	0	/	5	0	1	6	0	Alive
PC-8	M	59	H	PDAC	IV	22.18	0	0	0	0	0	0	0	/	3	1	0	4	0	6.9	0	0	0	0	0	0	0	/	3	1	0	4	1	Died
PC-9	M	50	BT	PDAC	IV	28068	0	0	0	0	0	0	0	/	3	1	5	9	0	78,182	0	0	0	0	0	0	0	/	0	0	146	146	5	Died
PC-10	F	70	BT	PDAC	IV	3954	0	0	0	0	0	0	0	/	5	0	3	8	2	11,356	0	0	0	0	0	0	0	/	5	0	7	12	1	Died
PC-11	F	51	H	PDAC	IV	132,070	0	0	0	0	0	0	0	/	9	0	2	11	1	173,664	0	0	0	0	0	0	0	/	4	0	4	8	1	Died
PC-12	M	64	H	PDAC	IV	71,019	0	0	0	0	0	0	0	/	0	1	1	2	0	73,780	0	0	0	0	0	0	0	/	3	1	0	4	1	Died
PC-13	M	59	H	PDAC	IV	4925	0	0	0	0	0	0	0	/	1	0	0	1	0															Died
PC-14	M	65	H	PDAC	IV	2222	0	0	0	0	0	0	0	/	0	1	0	1	0															Died
PC-15	M	74	BT	PDAC	IV	28.33	0	0	0	0	0	0	0	/	1	1	0	2	0															Lost
PC-16	M	76	BT	PDAC	IV	15.13	0	0	0	0	0	0	0	/	2	0	0	2	0															Died
PC-17	M	50	H	PDAC	IV	4423	0	0	0	0	0	0	0	/	0	2	1	3	0															Died
PC-18	M	75	H	PDAC	IV	4.25	0	0	0	0	0	0	0	/	3	0	0	3	0															Died
PC-19	F	48	H	PDAC	IV	10,313	0	0	0	0	0	0	0	/	3	0	0	3	0															Died
PC-20	F	48	H	PDAC	IV	442	0	0	0	0	0	0	0	/	1	1	1	3	0															Died
PC-21	M	39	BT	PDAC	IV	80,749	0	0	0	0	0	0	0	/	3	0	0	3	0															Died
PC-22	M	76	BT	PDAC	IV	19,764	0	0	0	0	0	0	0	/	4	0	1	5	0															Died
PC-23	M	36	H	PDAC	IV	6.09	0	0	0	0	0	0	0	/	2	4	2	8	0															Died
PC-24	F	73	H	PDAC	IV	9038	0	0	0	0	0	0	0	/	6	5	11	22	4															Alive
PC-25	F	64	H	PDAC	III	187.9	0	0	0	0	0	0	0	/	0	1	0	1	0															Died
PC-26	M	70	BT	PDAC	III	39.40	0	0	0	0	0	0	0	/	1	0	0	1	0															Alive
PC-27	M	50	H	PDAC	III	12.50	0	0	0	0	0	0	0	/	1	0	1	2	0															Alive
PC-28	F	21	H	PDAC	III	4.68	0	0	0	0	0	0	1	/	1	1	1	4	0															Died
PC-29	M	72	BT	PDAC	III	848.4	0	0	0	0	0	0	0	/	3	1	0	4	0															Died
PC-30	F	58	H	PDAC	II	2956	0	0	0	0	0	0	0	/	2	0	0	2	0															Died
PC-31	F	71	H	PDAC	II	18,834	0	0	0	0	0	0	0	/	0	0	1	1	0															Died
PC-32	M	56	BT	PDAC	II	124.5	0	0	0	0	0	0	0	/	3	0	0	3	0															Died
PC-33	F	68	BT	PDAC	II	124.20	0	0	0	0	0	0	0	/	1	0	1	2	0															Alive
PC-34	F	58	H	PDAC	II	1393	0	0	0	0	0	0	0	/	2	1	0	3	0															Lost
PC-35	M	62	H	PDAC	II	942.7	0	0	0	0	0	0	0	/	1	3	0	4	0															Alive
PC-36	M	71	H	PDAC	II	354.1	0	0	0	0	0	0	0	/	0	0	0	0	0															Died
PC-37	M	65	BT	PDAC	I	262.1	0	0	0	0	0	0	0	/	3	0	0	3	0															Died
PC-38	F	59	BT	PDAC	I	124.5	0	0	0	0	0	0	0	/	2	1	0	3	0															Lost
PC-39	F	64	H	PDAC	I	2088	0	0	0	0	0	0	0	/	0	0	0	0	0															Died
PC-40	F	49	H	PDAC	I	90.52	0	0	0	0	0	0	0	/	1	0	1	2	0															Died
BTP-1	F			PCN			0	0	0	0	0	0	0	/	0	2	0	2	0															
BTP-2	M			AIP			0	0	0	0	0	0	0	/	0	0	0	0	0															
BTP-3	M			PCN			0	0	0	0	0	0	0	/	0	1	0	1	0															
BTP-4	M			IPMN			0	0	0	0	0	0	0	/	0	0	0	0	0															
BTP-5	F			IPMN			0	0	0	0	1	1	0	/	0	0	1	1	0															
BTP-6	F			PCN			0	0	0	0	0	0	0	/	1	0	1	2	0															
BTP-7	M			IPMN			0	0	0	0	1	1	0	/	1	0	0	1	0															
BTP-8	M			AC			0	0	0	0	0	0	0	/	0	1	1	2	0															
HC-1							0	0	0	0	0	0	0	/	0	0	0	0	0															
HC-2							0	0	0	0	0	0	0	/	0	0	1	2	0															
HC-3							0	0	0	0	0	0	0	/	0	0	0	0	0															
HC-4							0	0	0	0	0	0	0	/	0	0	0	0	0															
HC-5							0	0	0	0	0	0	0	/	0	1	0	1	0															
HC-6							0	0	0	0	0	0	0	/	0	0	0	0	0															
HC-7							0	0	0	0	0	0	0	/	0	0	0	0	0															
HC-8							0	0	0	0	0	0	0	/	0	0	0	0	0															
HC-9							0	0	0	0	0	0	0	/	0	0	0	0	0															
HC-10							0	0	0	0	0	0	0	/	0	0	0	0	0															
HC-11							0	0	0	0	0	0	0	/	0	0	0	0	0															
HC-12							0	0	0	0	0	0	0	/	0	0	0	0	0															
HC-13							0	0	0	0	0	0	0	/	0	0	0	0	0															
HC-14							0	0	0	0	0	0	0	/	0	0	0	0	0															
HC-15							0	0	0	0	0	0	0	/	0	0	1	1	0															
HC-16							0	0	0	0	0	0	0	/	0	0	0	0	0															
HC-17							0	0	0	0	0	0	0	/	0	0	0	0	0															
HC-18							0	0	0	0	0	0	0	/	0	1	0	1	0															
HC-19							0	0	0	0	0	0	0	/	0	1	1	2	0															
HC-20							0	0	0	0	0	0	0	/	2	0	0	2	0															
HC-21							0	0	0	0	0	0	0	/	0	0	0	0	0															
HC-22							0	0	0	0	0	0	0	/	1	0	0	1	0															
HC-23							0	0	0	0	0	0	0	/	0	0	1	1	0															
HC-24							0	0	0	0	0	0	0	/	0	0	0	0	0															
HC-25							0	0	0	0	0	0	0	/	0	0	0	0	0															
HC-26							0	0	0	0	0	0	0	/	0	0	0	0	0															
HC-27							0	0	0	0	0	0	0	/	0	1	0	1	0															
HC-28							0	0	0	0	0	0	0	/	0	0	1	1	0															
HC-29							0	0	0	0	0	0	0	/	0	0	0	0	0															
HC-30							0	0	0	0	0	0	0	/	1	0	0	1	0															
HC-31							0	0	0	0	0	0	0	/	0	0	0	0	0															
HC-32							0	0	0	0	0	0	0	/	0	0	0	0	0															
HC-33							0	0	0	0	0	0	0	/	0	0	0	0	0															
HC-34							0	0	0	0	0	0	0	/	1	1	0	2	0															
HC-35							0	0	0	0	0	0	0	/	0	0	0	0	0															

F, Female; M, Male; H, Head; BT, Body and Tail; PC, Pancreatic cancer; BTP, Benign tumors of the pancreas; HC, Healthy control; PDAC, Pancreatic ductal adenocarcinoma; PCN, Pancreatic cystic neoplasms; AIP, Autoimmune pancreatitis; IPMN, pancreatic intraductal papillary mucinous neoplasms; AC, Acute pancreatitis; CTC: circulating tumor cells. CEP-8: chromosome enumeration probes 8; DAPI: 4′,6-diamidino-2-phenylindole; CK18: cytokeratins 18; CD45: cluster of differentiation 45; EpCAM: epithelial cell adhesion molecule; CA 19-9: carbohydrate antigen 19-9. /: Not included in CTC.

**Table 2:** Tumor cells from five cell lines found by the technologists and electronic microscope.

Cell Line	Cells Found by Technologist	Cells Found by the Electronic Microscope			Average
		Cycle 1	Cycle 2	Cycle 3	
Bxpc-3	0	0	0	0	0
	6	6	6	6	6
	12	12	12	12	12
	186	193	191	191	191
PANC-1	0	0	0	0	0
	5	5	5	5	5
	10	10	10	10	10
	172	186	182	178	182
A549	11	11	11	10	10
	32	32	32	31	31
	118	120	118	119	119
SW480	5	5	5	5	5
	34	34	34	33	33
	98	98	98	96	97
SK-BR3	7	7	7	7	7
	41	43	43	43	43
	140	145	143	143	143

**Table 3:** CTM counts and CA19-9 in five PDAC patients.

Patients Codes	Stage	Before Chemotherapy		After 3 Weeks	
		The Number of CTMs	CA19-9 Value (U/mL)	The Number of CTMs	CA19-9 Value (U/mL)
PC-8	IV	0	22.18	1	6.9
PC-9	IV	0	13,162	5	78,182
PC-10	IV	2	3954	1	11,356
PC-11	IV	1	132,070	1	173,664
PC-12	IV	0	71,019	1	73,780

## Supplementary Materials

Supplementary materials can be found at www.mdpi.com/1422-0067/18/4/622/s1.

## Abbreviations

CTCs	Circulating tumor cells
CTMs	Circulating tumor microembolus
PC	Pancreatic cancer
BTP	Benign tumors of the pancreas
HC	Healthy control
PDAC	Pancreatic ductal adenocarcinoma
PNET	Pancreatic neuroendocrine tumors
PCN	Pancreatic cystic neoplasms
AIP	Autoimmune pancreatitis
IPMN	Pancreatic intraductal papillary mucinous neoplasms
AC	Acute pancreatitis
CA19-9	Carbohydrate antigen 19-9
ASCO	American Society of Clinical Oncology
