# Supplementary material for: Detection of Circulating Tumor Cells Using Negative Enrichment Immunofluorescence and an In Situ Hybridization System in Pancreatic Cancer
Source: Int J Mol Sci. 2017 Mar 23;18(4):622. doi: 10.3390/ijms18040622 (PMC5412265; doi:10.3390/ijms18040622)
Supplement: Supplementary file 1 [file ijms-18-00622-s001.pdf]

Supplementary Data

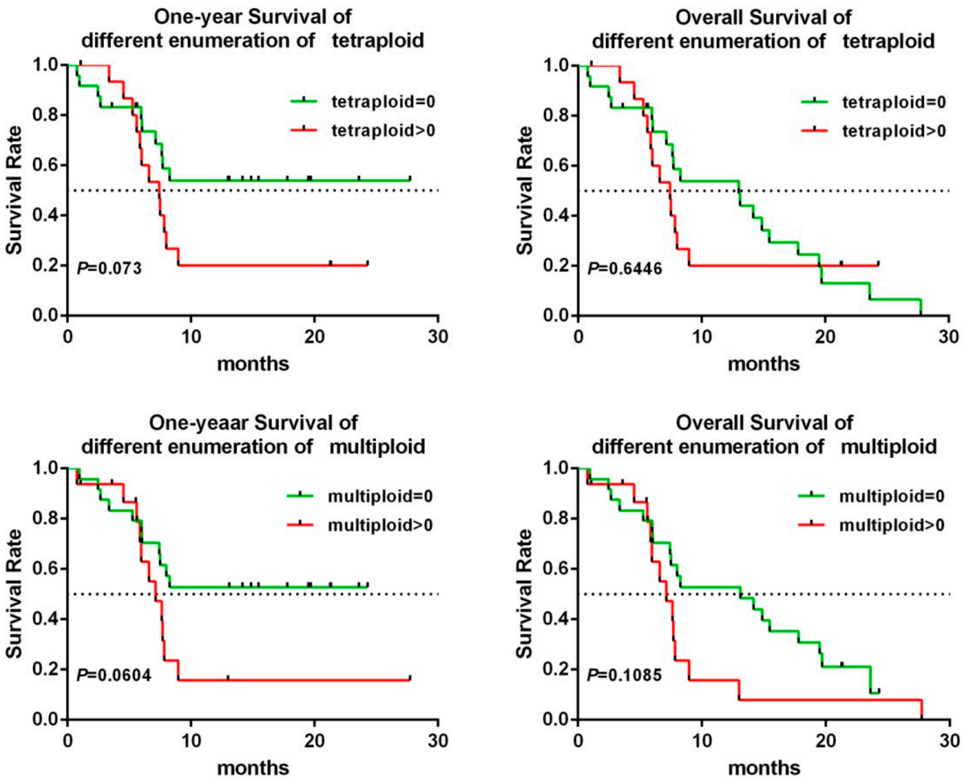

Supplementary Figure S1. Correlation between aneuploidy (tetraploid and multiploid CTCs) and the clinical prognosis. Overall Survival and one-year survival are irrelevant to the number of tetraploid and multiploid CTCs. CTCs: circulating tumor cells.

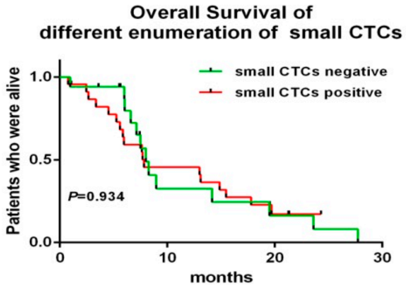

Supplementary Figure S2. Overall survival with different numbers of small CTCs. There is no relationship between overall survival and the number of small CTCs (hazard ratio for death, 0.9713; 95% CI 0.4757 to 1.975, *P*=0.934).

Supplementary Table S1. Clinical characteristics of the pancreatic cancer patients.

Variable	NO.	No.of patients(%)		*P* value
		CTCs number≥2/7.5ml	CTCs number＜2/7.5ml	
Total patients	40	31（77.5%）	9（22.5%）	
Age				0.142
≥65	14	9（64.3%）	5（35.7%）	
＜65	26	22（84.6%）	4（15.4%）	
Gender				0.769
male	25	19（76%）	6（24%）	
female	15	12（80%）	3（20%）	
Tumor Stage				0.656
I-II	11	8（72.7%）	3（27.3%）	
III-IV	29	23（79.3%）	6（20.7%）	
Lymph nodes				0.835
N0	19	15（78.9%）	4（21.1%）	
N1	21	16（76.2%）	5（23.8%）	
Metastasis				0.368
M0	17	12（70.6%）	5（29.4%）	
M1	23	19（82.6%）	4（17.4%）	
Pancreatic tumor location				0.804
Head	28	22（78.6%）	6（21.4%）	
Body and tail	12	9（75%）	3（25%）	
CA199（umol/L)				0.142
＞37	31	23（74.2%）	8（25.8%）	
≤37	9	8（88.9%）	1（11.1%）	
